# Reduced economic disparity in biologics use for psoriasis after introducing the reducing copayment program

**DOI:** 10.1038/s41598-024-54447-5

**Published:** 2024-02-20

**Authors:** Hyemin Jung, Seong Rae Kim, Soo Ick Cho, Seong Jin Jo

**Affiliations:** 1 Department of Health Policy and Management, Seoul National University College of Medicine, Seoul, South Korea; 2 Department of Education and Human Resource Developement, Seoul National University Hospital, Seoul, South Korea; 3 Department of Dermatology, Seoul National University College of Medicine, Seoul, South Korea; 4 Lunit, Seoul, South Korea

**Keywords:** Psoriasis, Biologics, Insurance coverage, Reducing copayment program, Drug survival, Economic disparity, Skin diseases, Health policy, Health care economics

## Abstract

Biologics for psoriasis are efficient and safe, but very expensive. We investigated the association of the reducing copayment program (RCP) with changes in biologics use patterns depending on the income levels of patients with moderate-to-severe psoriasis. This nationwide cohort study included patients identified as having moderate-to-severe psoriasis between 2014 and 2020. Logistic regression models were used to estimate the odds ratio for the use of biologics according to income levels. Among 57,139 patients with moderate-to-severe psoriasis, 3464 (6.1%) used biologics for psoriasis from 2014 to 2020. After the introduction of RCP in 2017, the proportion of patients with moderate-to-severe psoriasis using biologics rapidly increased from 5.0% in 2016 to 19.2% in 2020; the increase was more remarkable in patients with the lowest or mid-low income compared to those with Medical Aid. Drug survival of biologics was higher in patients with the highest income before the RCP, but became comparable between those with high and low incomes after RCP introduction. The introduction of RCP was associated with an increased use of biologics in patients with moderate-to-severe psoriasis of all income levels; however, the effect was more pronounced in low-income patients. The RCP may contribute to alleviating the disparity in access to biologics.

## Introduction

Psoriasis is a common chronic inflammatory skin disease with characteristic erythematous scaly plaque^1,2^. Psoriasis may cause social stigmatization and degrade the patients’ quality of life^3,4^. Recently, multiple studies have reported that psoriasis ^1^is associated with not only previously well-known comorbidities—such as psoriatic arthritis, Crohn's disease, and uveitis—but also other chronic diseases, including cardiovascular disease, hypertension, diabetes, hyperlipidemia, and even psychiatric comorbidities^5–7^. Therefore, psoriasis is considered a systemic disease with a fairly high burden.

With remarkable recent accomplishments in understanding of the immunologic pathogenesis of psoriasis, the paradigm of psoriasis treatment has changed with the widespread introduction of biologics^8,9^. Various biologics for psoriasis are superior in efficacy and safety to conventional systemic treatment, such as cyclosporine, methotrexate, and acitretin. With biologics, nearly 90% of patients with psoriasis achieved 75% improvement in Psoriasis Area and Severity Index (PASI-75), and over 70% achieved PASI-90^8,9^. Furthermore, biologics may also alleviate the comorbidities of psoriasis^10^. Both the Joint American Academy of Dermatology (AAD)–National Psoriasis Foundation (NPF) and the European guidelines recommend biologics for patients with severe psoriasis refractory to conventional treatment^11^.

However, an obstacle to the accessibility of biologics is the high cost. In the United States, according to a recent study reporting cost-effectiveness analyses of biologics from 2017, the total annual costs of a health plan per patient for adalimumab, secukinumab, and ustekinumab were evaluated at $51,246, $57,510, and $57,013, respectively. These expenses were considerably more financially burdensome to patients with psoriasis than the cost of systemic therapies^12,13^. Indeed, the high cost of biologics may remarkably reduce their accessibility, especially for patients with financial difficulties^14^.

In South Korea, 97% of Koreans are covered by the National Health Insurance Service (NHIS) with a wide range of compulsory health insurance, and the remaining 3% who are socio-economically vulnerable are covered by Medical Aid (MA). NHIS beneficiaries pay an average of 30–60% of a total of medical expenses as copayment, while MA beneficiaries pay extremely low copayment. Those with rare incurable diseases are eligible to enroll in the reducing copayment program (RCP) to mitigate the burden of expensive medication and pay only 10% of the total medical expenses as copayment.

As part of the Korean government's policy to strengthen health insurance coverage, patients with moderate-to-severe psoriasis who were refractory to at least 6 months of conventional treatment were allowed to enroll in the RCP from June 2017, which contributed to a 90% reduction in medical treatment costs. Before June 2017, these patients had to pay an average of $6000 as copayment per year for using biologics; however, after the introduction of RCP, they only had to pay an average of $1000 per year.

To date, while there are several studies investigating the relationship between cost sharing and drug utilization^15,16^, there have been few studies examining whether medical policies or health insurance systems contribute to alleviating economic disparities in drug utilization. Furthermore, few studies have evaluated the impact of medical policies or health insurance systems on the treatment patterns of patients with moderate-to-severe psoriasis. Moreover, the limited studies conducted on psoriasis are predominantly from Western countries, and there is a scarcity of research conducted in Asian^15^. In this study, we aimed to examine the impact of RCP on the accessibility of biologics in patients with moderate-to-severe psoriasis according to their income levels.

## Results

### General characteristics of the patient with moderate-to-severe psoriasis

A total of 57,139 patients with moderate-to-severe psoriasis were identified between 2014 and 2020, of whom 34,760 (60.8%) were men (Table 1). The number of patients with moderate-to-severe psoriasis who used biologics has increased rapidly since 2017, when the RCP was introduced by NHIS for the patients with intractable psoriasis. As a result, the proportion of biologics use increased from 5.0% in patients with moderate-to-severe psoriasis in 2016 to 19.2% in 2020. As of December 2020, 2327 patients with moderate-to-severe psoriasis were enrolled in the RCP. Among them, 97.2% (2261/2327) used biologics, while only 2.2% (1203/54,812) of the RCP-unenrolled patients used biologics.

**Table 1:** General characteristics of the patient with moderate-to-severe psoriasis.

Variables	Whole period (2014–2020)	By year						
		2014	2015	2016	2017	2018	2019	2020
	N (%)	N (%)	N (%)	N (%)	N (%)	N (%)	N (%)	N (%)
Total	57,139	13,801	14,658	15,691	16,289	16,844	17,646	16,294
Sex								
Male	34,760 (60.8)	8824 (63.9)	9220 (62.9)	9846 (62.7)	10,321 (63.4)	10,767 (63.9)	11,267 (63.9)	10,469 (64.3)
Female	22,379 (39.2)	4977 (36.1)	5438 (37.1)	5845 (37.3)	5968 (36.6)	6077 (36.1)	6379 (36.1)	5825 (35.7)
Age group								
20–29	6324 (11.1)	1099 (8.0)	1143 (7.8)	1279 (8.2)	1336 (8.2)	1440 (8.5)	1506 (8.5)	1459 (9.0)
30–39	9213 (16.1)	2274 (16.5)	2308 (15.7)	2437 (15.5)	2477 (15.2)	2522 (15.0)	2506 (14.2)	2251 (13.8)
40–49	12,192 (21.3)	3224 (23.4)	3297 (22.5)	3400 (21.7)	3620 (22.2)	3602 (21.4)	3699 (21.0)	3401 (20.9)
50–59	13,461 (23.6)	3633 (26.3)	3791 (25.9)	4006 (25.5)	4100 (25.2)	4195 (24.9)	4429 (25.1)	3989 (24.5)
60–69	9457 (16.6)	2051 (14.9)	2419 (16.5)	2725 (17.4)	2881 (17.7)	3069 (18.2)	3298 (18.7)	3185 (19.5)
80–79	4962 (8.7)	1237 (9.0)	1347 (9.2)	1462 (9.3)	1474 (9.0)	1540 (9.1)	1622 (9.2)	1515 (9.3)
80-	1530 (2.7)	283 (2.1)	353 (2.4)	382 (2.4)	401 (2.5)	476 (2.8)	586 (3.3)	494 (3.0)
Income								
Medical Aid	2291 (4.0)	589 (4.3)	635 (4.3)	693 (4.4)	709 (4.4)	738 (4.4)	797 (4.5)	744 (4.6)
Lowest^a^	11,605 (20.3)	2682 (19.4)	2802 (19.1)	3042 (19.4)	3213 (19.7)	3394 (20.1)	3763 (21.3)	3241 (19.9)
Mid-low^a^	11,785 (20.6)	2932 (21.2)	3072 (21.0)	3263 (20.8)	3263 (20)	3467 (20.6)	3498 (19.8)	3400 (20.9)
Mid-high^a^	14,745 (25.8)	3557 (25.8)	3811 (26.0)	3948 (25.2)	4220 (25.9)	4209 (25.0)	4362 (24.7)	3982 (24.4)
Highest^a^	16,713 (29.2)	4041 (29.3)	4338 (29.6)	4745 (30.2)	4884 (30.0)	5036 (29.9)	5226 (29.6)	4927 (30.2)
Residence								
Metropolitan	38,981 (68.2)	9273 (67.2)	9803 (66.9)	10,875 (69.3)	11,254 (69.1)	1152 (6.8)	12,126 (68.7)	11,459 (70.3)
Others	18,158 (31.8)	4528 (32.8)	4855 (33.1)	4,816 (30.7)	5,035 (30.9)	5192 (30.8)	5520 (31.3)	4835 (29.7)
Biologics use								
Yes	3464 (6.1)	444 (3.2)	554 (3.8)	787 (5.0)	1,062 (6.5)	1,607 (9.5)	2341 (13.3)	3123 (19.2)
No	53,675 (93.9)	13,357 (96.8)	14,104 (96.2)	14,904 (95)	15,227 (93.5)	15,237 (90.5)	15,305 (86.7)	13,171 (80.8)
RCP*								
Enrolled	2327 (4.1)	–	–	–	570 (3.5)	1039 (6.2)	1626 (9.2)	2249 (13.8)
Not enrolled	54,812 (95.9)	–	–	–	15,719 (96.5)	15,805 (93.8)	16,020 (90.8)	14,045 (86.2)
Mortality	445 (0.8)	60 (0.4)	57 (0.4)	56 (0.4)	64 (0.4)	70 (0.4)	70 (0.4)	68 (0.4)

^a^Four groups were classified according to income levels among NHIS beneficiaries.

*NHIS* National Health Insurance Service; *RCP* reducing copayment program; *N* the number of people.

### Rapid increase in the proportion of biologics users after the introduction of RCP

**Figure 1:**
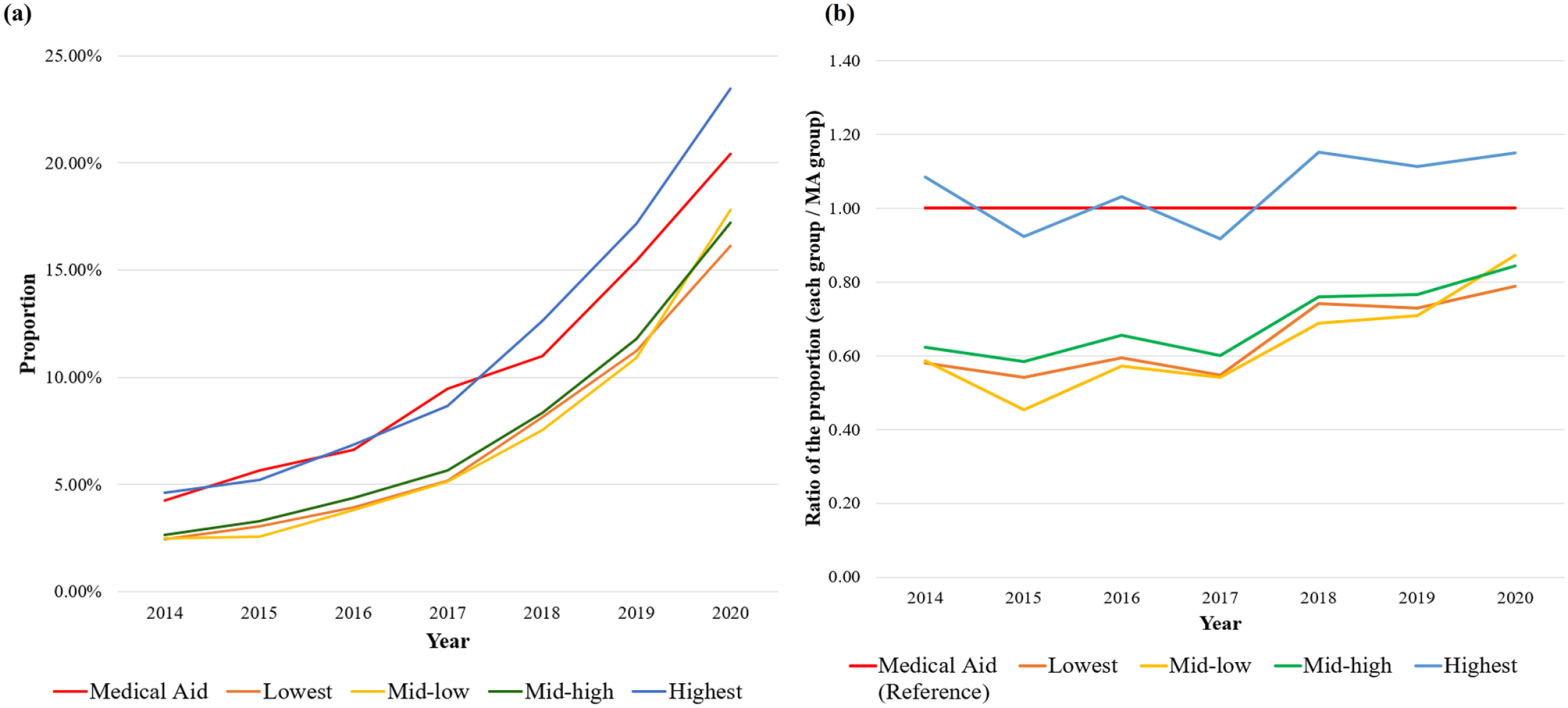
(**a**) The proportion of biologics users among the patients with moderate-to-severe psoriasis in each income group and (**b**) the ratio of the proportion of biologics users in each income group to that in the MA group. All groups, except MA, were classified according to income levels among NHIS beneficiaries. *MA* medical aid, *NHIS* National Health Insurance Service.

The proportions of biologics users among those with moderate-to-severe psoriasis are presented by income levels in Fig. 1a and Supplementary Table S1. In general, the proportions of biologics users increased in all income groups over time. The highest income groups demonstrated higher proportions of biologics user than the other income groups, except the MA group. Since the MA group was not affected by the RCP during the study period, we compared the proportion of biologics users in each income group to the proportion of those in the MA group in each year. As shown in Fig. 1b and Supplementary Table S2, the ratio of the proportion of biologics users in each income group to that in the MA group has steadily increased since 2017. The increases in this ratio were remarkable in the groups with the lowest income and mid-low income (0.55 [95% confidence interval [CI] 0.42–0.72] and 0.54 [95% CI 0.41–0.71] in 2017, respectively; 0.79 [95% CI 0.67–0.93] and 0.87 [95% CI 0.74–1.02] in 2020, respectively).

### Increase in the proportion of biologic users in the RCP-enrolled patients

We performed logistic regression analyses to identify the socioeconomic factors associated with the use of biologics (Supplementary Table S3). Patients who were men, young, and metropolitan residents were more likely to use biologics. In the entire cohort, patients with the lowest (adjusted odds ratio [aOR] 0.60, 95% CI 0.50–0.73), mid-low (aOR 0.59, 95% CI 0.49–0.71), mid-high (aOR 0.61, 95% CI 0.51–0.73), and highest (aOR 0.84, 95% CI 0.71–1.00) income were less likely to use biologics compared with those with MA. The subgroup analysis showed a consistent tendency in the RCP-unenrolled group (Table 2). However, this disparity in biologics use was improved in the RCP-enrolled groups, particularly in the highest (aOR 4.12, 95% CI 1.59–10.7) and the mid-high (aOR 3.62, 95% CI 1.32–9.98) income groups (Table 2).

**Table 2:** Socio-economic factors associated with the use of biologics in RCP-enrolled and RCP-unenrolled patients with moderate-to-severe psoriasis.

Variables	RCP enrolled (N = 2327)		Not enrolled (N = 54,812)	
	aOR (95% CI)^a^	*P* value	aOR (95% CI)^a^	*P* value
Sex				
Male	1.00 (reference)		1.00 (reference)	
Female	0.90 (0.54–1.52)	0.71	0.79 (0.70–0.89)	< 0.001
Age group				
20–29	1.00 (reference)		1.00 (reference)	
30–39	0.54 (0.15–1.95)	0.35	1.59 (1.27–1.99)	< 0.001
40–49	0.50 (0.14–1.77)	0.29	1.36 (1.09–1.69)	0.007
50–59	0.37 (0.11–1.29)	0.12	1.26 (1.01–1.56)	0.04
60–69	0.33 (0.09–1.23)	0.10	0.91 (0.71–1.16)	0.47
80–79	0.12 (0.03–0.48)	0.003	0.53 (0.38–0.74)	< 0.001
80-	0.04 (0.01–0.27)	< 0.001	0.44 (0.25–0.78)	0.004
Income group				
Medical Aid	1.00 (reference)		1.00 (reference)	
Lowest	2.35 (0.87–6.38)	0.09	0.34 (0.26–0.43)	< 0.001
Mid-low	2.33 (0.85–6.36)	0.10	0.32 (0.25–0.41)	< 0.001
Mid-high	3.62 (1.32–9.98)	0.01	0.30 (0.23–0.38)	< 0.001
Highest	4.12 (1.59–10.7)	0.004	0.47 (0.38–0.59)	< 0.001
Residence				
Metropolitan	1.00 (reference)		1.00 (reference)	
Others	0.48 (0.29–0.81)	0.006	0.57 (0.50–0.66)	< 0.001

*aOR* adjusted odds ratio, *CI* confidence interval, *RCP* reducing copayment program.

^a^aOR (95% CI) were derived by logistic regression analysis after adjusting for age, sex, income, and residence.

### Improved drug survival of biologics after the introduction of RCP

**Figure 2:**
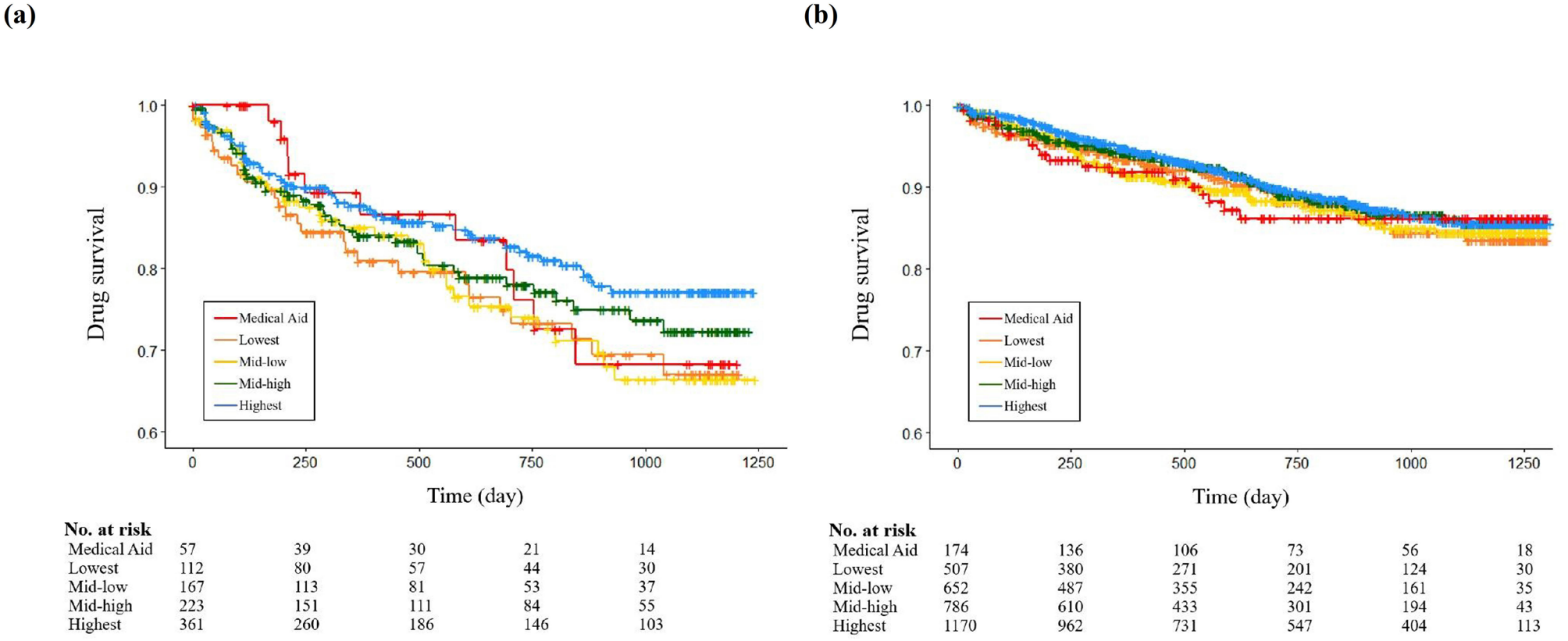
Drug survival of biologics for treating moderate-to-severe psoriasis according to income levels (**a**) before the introduction of the RCP (June 2017) and (**b**) after the introduction of the RCP. All groups, except MA, were classified according to income levels among NHIS beneficiaries. *RCP* reducing copayment program, *MA* medical aid, *NHIS* National Health Insurance Service, *No* number.

The drug survival of biologics for treating moderate-to-severe psoriasis according to income levels using the Kaplan–Meier plot is shown in Fig. 2. Before the introduction of the RCP in June 2017, the drug survival of biologics was higher in patients with the highest income than in those with the lowest income (*P* = 0.08, marginally attenuated; Supplementary Table S4). In contrast, after the introduction of the RCP, the drug survival of biologics was similar between patients with high and low incomes. Furthermore, drug survival increased in all income groups. As shown in Table 3, all patients in each income group, except those with MA, after the introduction of the RCP had a decreased risk of drug discontinuation compared with those before the introduction of the RCP (lowest income group: adjusted hazard ratio [aHR] 0.74, 95% CI 0.63–0.87; mid-low income group: aHR 0.75, 95% CI 0.65–0.86; mid-high income group: aHR 0.75, 95% CI 0.66–0.85; highest income group: aHR 0.80, 95% CI 0.72–0.89).

**Table 3:** Hazard ratios for drug discontinuation of biologics in patients with moderate-to-severe psoriasis.

	After the introduction of RCP (June 2017) vs before the introduction of RCP (Ref)			
	cHR (95% CI)	*P* value	aHR (95% CI)^a^	*P* value
Income group				
Medical Aid	0.81 (0.63–1.04)	0.09	0.79 (0.61–1.02)	0.07
Lowest	0.74 (0.64–0.87)	< 0.001	0.74 (0.63–0.87)	< 0.001
Mid-low	0.75 (0.66–0.86)	< 0.001	0.75 (0.65–0.86)	< 0.001
Mid-high	0.76 (0.67–0.86)	< 0.001	0.75 (0.66–0.85)	< 0.001
Highest	0.81 (0.73–0.90)	< 0.001	0.80 (0.72–0.89)	< 0.001

^a^aHR (95% CI) were derived by Cox hazards regressions analysis after adjusting for age, sex, and residence.

*cHR* crude hazard ratio, *aHR* adjusted hazard ratio, *CI* confidence interval, *RCP* reducing copayment program.

## Discussion

We identified that the number of biologics users for psoriasis has been increasing steadily each year. The introduction of the RCP policy has facilitated access to biologics for patients with moderate to severe psoriasis. In particular, an increase in the use of biologic agents depending on the RCP was more pronounced in patients with low incomes (the lowest or mid-low incomes) than in those with high incomes. Patients with the highest income had higher drug survival for biologics before the introduction of the RCP; however, after RCP was introduced, the difference in drug survival between patients with high and low incomes significantly diminished. This is the first study to show the impact of a special healthcare policy on patients with moderate-to-severe psoriasis in terms of access to biologics by the patients’ economic status.

Within several decades, outstanding achievements in understanding the etiology of psoriasis have contributed to the development of biologics for psoriasis^8,9^. The treatment paradigm for psoriasis has shifted from conventional to biologics therapies, as various conventional treatments for psoriasis are associated with side effects, organ toxicity, and inconvenience. Multiple studies have reported that biologics for psoriasis are effective, convenient, and safe and can be an alternative to conventional therapies^10,17–19^. However, their high costs may cause inequality in the treatment of psoriasis due to patients’ financial conditions ^20^. Indeed, we found that the proportion of biologics users among patients with moderate-to-severe psoriasis was much higher in patients with the highest income level and MA beneficiaries before the introduction of the RCP. It is notable that MA beneficiaries are socioeconomically vulnerable; thus, they are exempt from almost all medical expenses. This suggests that the cost of biologics is an obstacle for the use of biological agents.

After the introduction of the RCP in 2017, the use of biologics for psoriasis rapidly increased in both high- and low-income patients. These results are in line with those of previous studies reporting that the proportion of biologics users for several other refractory diseases, including refractory inflammatory bowel disease and rheumatoid arthritis, increased after insurance coverage expansion, such as the RCP^21,22^. In particular, compared with the proportion of biologics users in patients with MA, who are the least likely to be affected by the RCP introduction, the proportion of biologics users in groups with low income noticeably and steadily increased after the introduction of the RCP, implying that the mitigation of medical expense barriers through the RCP played an important role in the use of biologics. Furthermore, the proportion of biologics users among patients with the highest income also considerably increased, surpassing that of patients with MA after the RCP introduction in 2017. Patients with MA were more likely to use biologics among those who were not enrolled in the RCP, while those with high incomes (the highest and mid-high incomes) were more likely to use biologics among those enrolled in the RCP. This implies that medical expense barriers also affect the use of biologics for psoriasis, even in patients with a high income.

The trend of drug survival of biologics for the treatment of moderate-to-severe psoriasis has also changed noticeably since the introduction of the RCP. Compared with patients with low incomes, the drug survival of biologics was higher in those with the highest income before the introduction of the RCP, while the disparity in the drug survival of biologics by income levels was attenuated after the introduction of the RCP. In addition, the continuation of biologics was enhanced in all patients, except patients with MA who were least affected by the RCP. This suggests that the introduction of the RCP may contribute to prolonging overall drug survival in all patients with psoriasis and alleviating the disparity in long-term maintenance of biologics depending on income levels.

The relatively young patients (30s and 40s) were significantly more likely to use biologics for psoriasis. Considering that individuals in their 30s or 40s are mainly socioeconomically active, they could probably afford expensive biologics compared to those in other age groups. However, this trend was attenuated among those enrolled in the RCP, which indicates that the RCP has enable most age groups to use biologics regardless of their economic activity levels. We also found that patients with moderate-to-severe psoriasis living in metropolitan cities were more likely to use biologics than those living in other areas even after adjusting for income levels. According to previous studies, differences in the geographical distribution of medical facilities commonly depend on socioeconomic status and residential location^23,24^. Those living in metropolitan cities may be more likely to have better socioeconomic status and higher accessibility to medical facilities where biologics can be prescribed compared with those living in suburban or rural areas. These trends were maintained among those enrolled in the RCP. Therefore, further studies are needed to examine the disparity in the use of biologics by accessibility to medical facilities as well as medical costs.

Drug prescriptions may be influenced by changes in health behaviors over time, fluctuations in the national economic strength, the development of new drugs, or shifts in preferences for disease treatments, occurring at specific periods. Therefore, considering the potential impact of these factors during our study period may be necessary. According to a previous study investigating the usage patterns of biologics from 2014 to 2018 in patients with rheumatoid arthritis, who had been subject to RCP policies before psoriasis, the use of biologics in rheumatoid arthritis patients steadily increased during this period, but the rate of increase was modest. Particularly, unlike the significant surge in biologics use observed in our study's patients with psoriasis after 2017, the increase in biologics usage in patients with rheumatoid arthritis was not quite pronounced after 2017 ^25^. This suggests that the increased use of biologics in psoriasis patients after the introduction of RCP may be primarily influenced by the RCP policy itself, rather than broader socio-economic changes or other ancillary factors during the period.

This study has several limitations. First, a cause-and-effect relationship could not be guaranteed because of the cross-sectional nature of our study. Second, the generalizability might be limited due to the unique nature of the RCP in Korea. However, considering that many countries are implementing systems tailored to their socio-economic environments for patients with specific medical conditions and disabilities, as well as economically vulnerable populations, our study findings may be sufficiently applicable to other countries as well. Third, the release of new biologics in South Korea after 2017 may have, to a certain extent, contributed to the increase in biologics prescriptions. However, after the introduction of the RCP, not only did the drug survival of biologics become similar between patients with high and low incomes, but also the proportion of biologics users in the groups with the lowest income and mid-low income became much more pronounced compared to patients with MA or other income groups. Considering these factors, in addition to the general increase in prescriptions that new drugs can lead to, our findings indicate that the RCP has clearly reduced economic disparity in biologics use among moderate-to-severe psoriasis patients. Finally, additional clinical information, including the duration of disease or objective severity assessment indicators, was not collected owing to data limitations. However, we robustly evaluated psoriasis using validated methodologies from previous studies^26,27^.

Nevertheless, our study has multiple strengths. This is the first large cohort study to examine the influence of healthcare policies on biologics use in patients with moderate-to-severe psoriasis, stratified by income levels. Robust nationwide medical claims records from the NHIS support the verification of psoriasis and registration of RCP for psoriasis. Moreover, we performed analyses after adjusting for confounders, including age, sex, household income, and residence, which may have enhanced the reliability of the results. We also provided real-world evidence that the RCP may be of great help to patients with low incomes suffering from severe psoriasis by improving the accessibility to biologics.

In conclusion, our findings suggest that the high cost of biologics causes inequity in the treatment of psoriasis, which may be improved or resolved by the introduction of appropriate healthcare policies such as the RCP. The disparity in accessing biologic agents may lead to cumulative life course impairment in patients with economic vulnerability, including poor response to treatment and development of comorbidities. Therefore, intensive efforts by the government and medical communities are necessary to establish an efficient and socially protective medical insurance system for patients with psoriasis, particularly those who require the use of biologics.

## Methods

### Data sources

We used a customized research database provided by the NHIS of South Korea. The database contained information on beneficiaries, such as resident registration number, age, sex, place of residence, amount of monthly premium, and claims data for medical services covered by NHIS or MA. As most medical institutions in South Korea are under a fee-for-service system, they must submit for reimbursement what and how many medical services they provide. A retrospective cohort study was conducted by linking all medical claims data to the resident registration number of each beneficiary.

This study was reviewed and approved by the Institutional Review Board of Seoul National University Hospital and the NHIS (IRB No. 2104-041-1209 and NHIS-2021-1-596). All claims data were de-identified. As the NHIS cohort database follows strict confidentiality protocols by anonymizing data, the need for obtaining informed consent was waived. All methods and investigations were performed in accordance with the relevant guidelines and regulations.

### Study population

A psoriasis cohort was constructed from the patients’ principal and secondary diagnoses based on the claim data. Individuals who had the psoriasis code (L40 in ICD-10) on their claims between January 2014 and December 2020 were defined as patients with psoriasis and were included in the cohort. According to previous studies, moderate-to-severe psoriasis was defined as the cumulative use of systemic anti-psoriatic treatment for at least 4 weeks, such as cyclosporin, methotrexate, or retinoids, or the use of biologic agents, with International Classification of Diseases 10th Revision (ICD-10) code for psoriasis^26,27^. Patients who were prescribed biologics (infliximab, etanercept, adalimumab, ustekinumab, golimumab, secukinumab, ixekizumab, guselkumab, and risankizumab) more than once were defined as the biologics-treated group. Patients who received only systemic non-biologic agents (cyclosporine, methotrexate, and acitretin) for at least 4 weeks were defined as the non-biologics-treated group. Patients with mild psoriasis were excluded from this study because they are usually not prescribed biologics. Patients younger than 20 years were also excluded, as most biologics are only approved for adults.

In Korea, patients are enrolled in the RCP when they meet the following criteria: (1) histologically confirmed psoriasis, (2) PASI score of ≥ 10 and body surface area (BSA) ≥ 10% even after the end of previous treatments (systemic non-biologic therapy for ≥ 3 months and biweekly phototherapy for ≥ 3 months). Patients enrolled in the RCP pay only 10% of the total medical expenses for psoriasis. The RCP did not affect MA beneficiaries who pay extremely low copayment regardless of RCP enrollment.

### Definitions of variables

The date of psoriasis diagnosis was defined as the date of the first claim with a diagnosis code of psoriasis. Registration for the RCP for psoriasis was confirmed using the special registration code V280. The date of the RCP registration was defined as the date of the first claim with the code.

The age and income levels of the participants were assigned differently according to the range of analysis. When analyzing data by year, the age of the year was used. Age in 2017 was used for the entire cohort analysis. Income level was classified into five groups (highest, mid-high, mid-low, lowest, and MA) based on the monthly insurance premium, which was usually set by income. When analyzing by year, the income level for that year was used. For the whole cohort analysis, the income level in 2017 was used for patients diagnosed before 2018, while the income level in the diagnosis year was used for patients diagnosed from 2018 onwards.

### Statistical analysis

First, year-by-year descriptive analyses were performed to determine the annual trends in the use of biologics. Univariate and multivariate logistic regression analyses were performed for the entire cohort population to identify the socioeconomic factors associated with the use of biologics. In addition, subgroup analyses stratified by RCP enrollment were performed.

Drug survival analyses were conducted using the Kaplan–Meier method, presenting the probability of drug survival of biologics for patients with moderate-to-severe psoriasis by income level before and after the introduction of the RCP in June 2017. The log-rank test was performed to assess differences in drug survival distribution among the groups categorized by income levels. Cox proportional hazard models were used to evaluate the HRs and 95% CIs for drug discontinuation of biologics by income levels after the introduction of the RCP compared with that before the RCP. When performing the analyses before the introduction of the RCP, each patient was followed-up starting on the date of biologics therapy initiation and censored at the date of death or May 31, 2017, whichever came first. When conducting the analyses after the introduction of the RCP, each patient was followed-up starting on the date of biologics therapy initiation and censored at the date of death or 31 December 2020, whichever came first. During the observation period, the discontinuation of biologics therapy was defined as a period of over 180 days without treatment with biologics from the last date of biologics use.

All analyses were conducted using the SAS Enterprise Guide, version 7.1 (SAS Institute, Inc., Cary, NC, USA).

### Supplementary Information

Supplementary Tables.

## Data Availability

The data underlying this article cannot be shared publicly due to the privacy or ethical restrictions of individuals that participated in the study. However, the data can be provided from the corresponding author upon a reasonable request and with the approval of the NHIS.

## Abbreviations

BSA: Body surface area
MA: Medical Aid
NHIS: National Health Insurance Service
PASI: Psoriasis Area and Severity Index
RCP: Reducing copayment program
aHR: Adjusted hazard ratio
aOR: Adjusted odds ratio
CI: Confidence interval
ICD: International Classification of Diseases
IRB: Institutional Review Board
