# Supplementary material for: Reduced economic disparity in biologics use for psoriasis after introducing the reducing copayment program
Source: Sci Rep. 2024 Feb 20;14:4139. doi: 10.1038/s41598-024-54447-5 (PMC10876659; doi:10.1038/s41598-024-54447-5)
Supplement: Supplementary file 1 — Supplementary Tables. [file 41598_2024_54447_MOESM1_ESM.docx]

**SUPPLEMENTAL MATERIAL**

**Journal name**: Scientific reports

**Title**: Reduced economic disparity in biologics use for psoriasis after introducing the reducing copayment program

**Authors**: Hyemin Jung, Seong Rae Kim, Soo Ick Cho, Seong Jin Jo

**Corresponding author**:

Soo Ick Cho, MD, PhD

Lunit Inc., 374 Gangnam-daero, Gangnam-gu, 06241, Seoul, Republic of Korea

Tel: +82-2-2138-0827, Fax: +82-2-6919-2702, E-mail: [sooickcho@lunit.io](mailto:sooickcho@lunit.io)

Seong Jin Jo, MD, PhD

Department of Dermatology, Seoul National University College of Medicine, 103 Daehak-ro, Jongno-gu, 03080, Seoul, Republic of Korea

Tel: +82-2-2072-4916, Fax: +82-2-742-7344, E-mail: [sj.jo@snu.ac.kr](mailto:sj.jo@snu.ac.kr)

**Supplementary Table S1.** The number and proportion of patients who use biologics for psoriasis by income.

| **Income group** | **By year** | | | | | | |
| --- | --- | --- | --- | --- | --- | --- | --- |
|  | **2014** | **2015** | **2016** | **2017** | **2018** | **2019** | **2020** |
|  | **N (%)** | **N (%)** | **N (%)** | **N (%)** | **N (%)** | **N (%)** | **N (%)** |
| **Medical Aid** | 25 (4.2) | 36 (5.7) | 46 (6.6) | 67 (9.5) | 81 (11.0) | 123 (15.4) | 152 (20.4) |
| **Lowest** | 66 (2.5) | 86 (3.1) | 120 (3.9) | 166 (5.2) | 276 (8.1) | 423 (11.2) | 522 (16.1) |
| **Mid-low** | 73 (2.5) | 79 (2.6) | 124 (3.8) | 167 (5.1) | 262 (7.6) | 382 (10.9) | 606 (17.8) |
| **Mid-high** | 94 (2.6) | 126 (3.3) | 172 (4.4) | 239 (5.7) | 351 (8.3) | 515 (11.8) | 686 (17.2) |
| **Highest** | 186 (4.6) | 227 (5.2) | 325 (6.9) | 423 (8.7) | 637 (12.7) | 898 (17.2) | 1157 (23.5) |
| **Subtotal** | 444 (3.2) | 554 (3.8) | 787 (5.0) | 1062 (6.5) | 1607 (9.5) | 2341 (13.3) | 3123 (19.2) |
| **Total** | 13,801 | 14,658 | 15,691 | 16,289 | 16,844 | 17,646 | 16,294 |

*N* the number of people.

**Supplementary Table S2.** The ratio (95% confidence interval) of the proportion of biologics users in each income group to that in the Medical Aid group

| **Income group** | **By year** | | | | | | |
| --- | --- | --- | --- | --- | --- | --- | --- |
|  | **2014** | **2015** | **2016** | **2017** | **2018** | **2019** | **2020** |
| **Medical Aid** | 1.00 (Ref) | 1.00 (Ref) | 1.00 (Ref) | 1.00 (Ref) | 1.00 (Ref) | 1.00 (Ref) | 1.00 (Ref) |
| **Lowest** | 0.58 (0.37-0.91) | 0.54 (0.37-0.79) | 0.59 (0.43-0.83) | 0.55 (0.42-0.72) | 0.74 (0.59-0.94) | 0.73 (0.61-0.88) | 0.79 (0.67-0.93) |
| **Mid-low** | 0.59 (0.38-0.92) | 0.45 (0.31-0.67) | 0.57 (0.41-0.80) | 0.54 (0.41-0.71) | 0.69 (0.54-0.87) | 0.71 (0.59-0.85) | 0.87 (0.74-1.02) |
| **Mid-high** | 0.62 (0.40-0.96) | 0.58 (0.41-0.84) | 0.66 (0.48-0.90) | 0.60 (0.46-0.78) | 0.76 (0.60-0.95) | 0.77 (0.64-0.92) | 0.84 (0.72-0.99) |
| **Highest** | 1.08 (0.72-1.63) | 0.92 (0.66-1.30) | 1.03 (0.77-1.39) | 0.92 (0.72-1.17) | 1.15 (0.93-1.43) | 1.11 (0.94-1.32) | 1.15 (0.99-1.34) |

**Supplementary Table S3.** Factors associated with biologics use in moderate to severe psoriasis patients

| **Variables** |  | **cOR (95% CI)** | ***P* value** | **aOR (95% CI)^a^** | ***P* value** |
| --- | --- | --- | --- | --- | --- |
| **Sex** | **Male** | 1.00 (reference) |  | 1.00 (reference) |  |
|  | **Femal** | **0.73 (0.68-0.79)** | **<0.001** | **0.75 (0.70-0.81)** | **<0.001** |
| **Age group** | **20-29** | 1.00 (reference) |  | 1.00 (reference) |  |
|  | **30-39** | **1.60 (1.41-1.83)** | **<0.001** | **1.62 (1.42-1.84)** | **<0.001** |
|  | **40-49** | **1.45 (1.28-1.65)** | **<0.001** | **1.39 (1.22-1.58)** | **<0.001** |
|  | **50-59** | 1.08 (0.95-1.23) | 0.26 | 1.06 (0.93-1.20) | 0.42 |
|  | **60-69** | **0.78 (0.67-0.90)** | **<0.001** | **0.76 (0.65-0.87)** | **<0.001** |
|  | **80-79** | **0.45 (0.37-0.56)** | **<0.001** | **0.42 (0.35-0.52)** | **<0.001** |
|  | **80-** | **0.28 (0.19-0.43)** | **<0.001** | **0.28 (0.18-0.42)** | **<0.001** |
| **Income group** | **Medical Aid** | 1.00 (reference) |  | 1.00 (reference) |  |
|  | **Lowest**^b^ | **0.75 (0.63-0.90)** | **0.002** | **0.60 (0.50-0.73)** | **<0.001** |
|  | **Mid-low**^b^ | **0.77 (0.64-0.92)** | **0.005** | **0.59 (0.49-0.71)** | **<0.001** |
|  | **Mid-high**^b^ | **0.82 (0.69-0.98)** | **0.02** | **0.61 (0.51-0.73)** | **<0.001** |
|  | **Highest**^b^ | 1.04 (0.87-1.23) | 0.69 | **0.84 (0.71-1.00)** | **0.05** |
| **Residence** | **Metropolitan** | 1.00 (reference) |  | 1.00 (reference) |  |
|  | **Others** | **0.60 (0.55-0.65)** | **<0.001** | **0.63 (0.58-0.68)** | **<0.001** |

^a^aOR (95 % CI) were derived by logistic regression analysis after adjusting for age, sex, income, residence, and RCP enrollment.

^b^Four groups were classified according to income levels among NHIS beneficiaries.

*cOR* Crude odds ratio; *aOR* Adjusted odds ratio; *CI* Confidence interval.

**Supplementary Table S4.** Hazard ratios for drug discontinuation of biologics in patients with moderate-to-severe psoriasis by income levels.

|  | **Before the introduction of RCP** | | | |  | **After the introduction of RCP** | | | |
| --- | --- | --- | --- | --- | --- | --- | --- | --- | --- |
| **Income group** | **cHR (95% CI)** | ***P* value** | **aHR (95% CI)^a^** | ***P* value** |  | **cHR (95% CI)** | ***P* value** | **aHR (95% CI)^a^** | ***P* value** |
| **Lowest** | 1.00 (Ref) |  | 1.00 (Ref) |  |  | 1.00 (Ref) |  | 1.00 (Ref) |  |
| **Mid-low** | 0.97 (0.59-1.59) | 0.90 | 0.97 (0.59-1.60) | 0.90 |  | 1.00 (0.70-1.44) | 0.99 | 1.04 (0.72-1.50) | 0.84 |
| **Mid-high** | 0.82 (0.51-1.33) | 0.43 | 0.82 (0.51-1.33) | 0.42 |  | 0.87 (0.61-1.25) | 0.46 | 0.90 (0.63-1.28) | 0.55 |
| **Highest** | 0.66 (0.42-1.05) | 0.08 | 0.67 (0.43-1.06) | 0.08 |  | 0.85 (0.61-1.18) | 0.32 | 0.84 (0.61-1.18) | 0.32 |
| **Medical Aid** | 0.78 (0.39-1.58) | 0.50 | 0.79 (0.39-1.60) | 0.51 |  | 1.00 (0.59-1.69) | 0.99 | 0.94 (0.55-1.59) | 0.80 |

^a^aHR (95 % CI) were derived by Cox hazards regressions analysis after adjusting for age, sex, and residence.

*cHR* crude hazard ratio; *aHR* adjusted hazard ratio; *CI* confidence interval; *RCP* reducing copayment program
